# A Concurrent Exposure to Arsenic and Fluoride from Drinking Water in Chihuahua, Mexico

**DOI:** 10.3390/ijerph120504587

**Published:** 2015-04-24

**Authors:** Carmen González-Horta, Lourdes Ballinas-Casarrubias, Blanca Sánchez-Ramírez, María C. Ishida, Angel Barrera-Hernández, Daniela Gutiérrez-Torres, Olga L. Zacarias, R. Jesse Saunders, Zuzana Drobná, Michelle A. Mendez, Gonzalo García-Vargas, Dana Loomis, Miroslav Stýblo, Luz M. Del Razo

**Affiliations:** 1 Facultad de Ciencias Químicas, Universidad Autónoma de Chihuahua, Chihuahua 31125, Mexico; E-Mails: mcgonzalezhorta@hotmail.com (C.G.-H.); lourdes.ballinas@gmail.com (L.B.-C.); bsanche@uach.mx (B.S.-R.); mariacishida@gmail.com (M.C.I.); mc.danielagutierrez@gmail.com (D.G.-T.); olga.zacarias@gmail.com (O.L.Z.); 2 Departamento de Toxicología, Centro de Investigación y de Estudios Avanzados del Instituto Politécnico Nacional (Cinvestav-IPN), México D. F. 07360, Mexico; E-Mail: darkgray@cinvestav.mx; 3 Department of Nutrition, Gillings School of Global Public Health, University of North Carolina at Chapel Hill, Chapel Hill, NC 27599-7461, USA; E-Mails: jesse.saunders@unc.edu (R.J.S.); zuzana_drobna@med.unc.edu (Z.D.); mmendez@email.unc.edu (M.A.M.); miroslav_styblo@med.unc.edu (M.S.); 4 Facultad de Medicina, Universidad Juárez del Estado de Durango (UJED), Gómez Palacio, Durango 35050, Mexico; E-Mail: ggarcia.vargas@gmail.com; 5 IARC Monographs Section, IARC/WHO, Lyon Cedex 69372, France; E-Mail: loomisd@iarc.fr

**Keywords:** adults, arsenic, drinking water, fluoride, groundwater

## Abstract

Inorganic arsenic (iAs) and fluoride (F^−^) are naturally occurring drinking water contaminants. However, co-exposure to these contaminants and its effects on human health are understudied. The goal of this study was examined exposures to iAs and F^−^ in Chihuahua, Mexico, where exposure to iAs in drinking water has been associated with adverse health effects. All 1119 eligible Chihuahua residents (>18 years) provided a sample of drinking water and spot urine samples. iAs and F^−^ concentrations in water samples ranged from 0.1 to 419.8 µg As/L and from 0.05 to 11.8 mg F^−^/L. Urinary arsenic (U-tAs) and urinary F^−^ (U-F^−^) levels ranged from 0.5 to 467.9 ng As/mL and from 0.1 to 14.4 µg F^−^/mL. A strong positive correlation was found between iAs and F^−^ concentrations in drinking water (*r_s_* = 0.741). Similarly, U-tAs levels correlated positively with U-F^−^ concentrations (*r_s_* = 0.633). These results show that Chihuahua residents exposed to high iAs concentrations in drinking water are also exposed to high levels of F^−^, raising questions about possible contribution of F^−^ exposure to the adverse effects that have so far been attributed only to iAs exposure. Thus, investigation of possible interactions between iAs and F^−^ exposures and its related health risks deserves immediate attention.

## 1. Introduction

Groundwater is a significant source for potable water for millions of people worldwide. Some of the aquifers are naturally enriched with inorganic arsenic (iAs) and/or fluoride (F^−^) at concentrations that may be hazardous to human health [1,2,3]. An excess of iAs and F^−^ in groundwater has been reported in many “hot spot” geographic areas in Latin America (e.g., Argentina, Bolivia, Chile, Colombia, Mexico, Peru) [1], Asia (e.g., China, India, Japan, Korea, Malaysia, Pakistan) [4], and Africa (Ethiopia, Ghana, Nigeria, Tanzania) [5,6]. Here, both iAs and F^−^ levels frequently exceed the corresponding World Health Organization (WHO) maximum contaminant level (MCL) of 10 µg As/L and 1.5 mg F^−^/L [7,8]. Although the U.S. Environmental Protection Agency set the MCL and secondary MCL values for F^−^ at 4 mg/L and 2 mg/L, respectively, the U.S. Department of Health and Human Services has recommended the value of 0.7 mg/L [9]. In comparison, the recommended range of F^−^ levels for the maximum protection from dental caries is thought to be 0.5–1 mg/L [10]. 

Generally, the occurrence of elevated concentrations of naturally occurring iAs or F^−^ in drinking water is a result of geothermal activities, mineral (e.g., arsenopyrite, fluorite, fluorapatite) dissolution, or deposition and weathering of atmospheric volcanic particles. Human exposure to F^−^ is also associated with the use of fluoridated dental products such as toothpastes and mouthwashes, with application of chemical fertilizers in agricultural areas, liquid waste from industrial sources, as well as with the fluorination of drinking water supplies in some countries and regions aiming to reduce the tooth decay [4,11]. Current data suggest that the excess of either iAs or F^−^ in groundwater can adversely affect health of millions of people worldwide. 

Arsenic is listed as the highest priority environmental contaminant by the Agency for Toxic Substances and Disease Registry [11]. The International Agency for Research on Cancer has classified iAs as a group 1 human carcinogen [12]. Chronic ingestion of drinking water with elevated iAs concentrations (≥500 µgAs/day) has been linked to complex toxicity syndrome generally known as arsenicosis. This syndrome is characterized by multiple disorders, including a variety of skin lesions (hypo- or hyper-pigmentation or keratosis) that can occur already after several years of exposure and which usually are the first visible signs of chronic iAs toxicity [8]. There is also strong evidence that chronic iAs ingestion increases risk of cardiovascular diseases [13], diabetes mellitus or impaired cognitive function in early childhood [14,15]. In comparison, chronic exposure to elevated F^−^ levels in drinking water (>8 mgF^−^/day) can lead to development of dental fluorosis characterized primarily by mottling of tooth enamel [4]. Additional risks of increased F^−^ exposure are known; the most significant are effects on bone cells (both osteoblasts and osteoclasts) causing the development of skeletal fluorosis. It is now also recognized that chronic F^−^ exposure affects cells in soft tissues, including renal, endothelial, gonadal, and neurological cells, causing functional and structural damage [16]. 

Although the co-occurrence of high iAs and F^−^ levels in drinking water has been reported in many geographical regions, only a few studies have used biomarkers to better characterize human exposures. Urinary arsenic (U-tAs) and urinary F^−^ (U-F^−^) are generally recognized as biomarkers of the exposure to iAs and F^−^ [17,18]. In this study, urinary As and F^−^ excretion were examined in subjects recruited among residents of Chihuahua, the area of Mexico which is known for concurrent high F^−^ and iAs in ground water [1] and where iAs exposure has been linked to adverse health effects [19,20].

## 2. Experimental Section 

### 2.1. Study Area

The study area in the Central-South region of Chihuahua, northern Mexico, is located between 26°41' and 29°20' latitude N and between 103°54' and 106°33' longitude W. This area includes the city of Chihuahua and small towns and settlements in 13 municipalities (Aldama, Camargo, Chihuahua, Coronado, Delicias, Jiménez, Julimes, La Cruz, Meoqui, Rosales, San Francisco de Conchos, Saucillo, and Satevó) with mainly agricultural and farming communities. The climate here is arid and semi-arid. The mean altitude is 1263 m, the temperature ranges from −10.1 °C to 41.7 °C with 300–400 mm average annual rainfall [21]. The mean depth of underground water is 110 m, ranging from 4 to 300 m. Many of the settlements have water purification systems; however, because of a limited maintenance or inconvenient access, many local residents use iAs contaminated water. 

### 2.2. Study Participants 

The study design was approved by the Institutional Review Boards of Cinvestav-IPN, Mexico City, Mexico and University of North Carolina at Chapel Hill, North Carolina, USA. The study subjects were recruited in household visits between 2008 and 2012, in base of information of current and historical levels of iAs and F^−^ in local drinking supplies [21]. A total of 1119 adult (18–90 years) Chihuahua residents, 370 men and 749 women, participated in this study. Only the subjects with a minimum of 5-year residency in the study area and without occupational exposure to iAs or F^−^ were recruited. Preference was given to those residents who did not use purified water for drinking and cooking. Both oral and written informed consents were obtained from all participants before admission to the study. A questionnaire was administered to all participants at the time of admission to collect demographic and residential data and information on occupation, medical history and drinking water sources. Pregnant women, alcoholics, and individuals with chronic or acute diseases of the urinary tract were excluded from participation in the study.

### 2.3. Tube Well Water Sample Collection

Four hundred forty five well water samples from 94 major rural localities of Chihuahua were collected during 2008 to 2012. Tube well water sampled represent 10% of the tube wells from the 13 municipalities of this study [21]. Three to six water samples in each locality were collected into new plastic bottles from tube wells that served as the primary sources of water for local households. After collection, the samples were stored at −20 °C and later transported on dry ice to Cinvestav-IPN (Mexico City) for As and F^−^ analysis.

### 2.4. Drinking Water and Urine Collection

Drinking water and spot urine samples were collected between 2008 and 2012. The samples of water typically used for drinking were collected in subjects’ homes and were kept frozen until analysis. Because the household water comes from the local wells and is supplied through municipal water networks to the subjects’ homes, the samples of well and household water should contain the same or similar levels of F^−^ and As. The urine samples were collected at Chihuahua University during morning hours after overnight fasting. Spot-urine samples were collected in clean disposable urine collection cups and immediately transferred to 15-mL polyethylene tubes (3 tubes per urine sample). All samples were frozen within 1–2 h after collection and stored at −80 °C at Chihuahua University. 

### 2.5. Determination of Arsenic and Fluoride Concentrations in Water

Arsenic was measured in acid digested samples by hydride generation-atomic fluorescence spectrometry (HG-AFS) [22] in Cinvestav-IPN, using a PSA 10.055 Millenium Excalibur continuous flow system (PS Analytical, Orpink, UK). Briefly, calibration curves were prepared using standard solutions of iAsIII at final concentrations of 1 to 10 µgAs/L. The water samples (1 mL each) were mixed with 3 mL of concentrated hydrochloric acid. The acidified samples were treated with 0.2 mL of 0.3% (m/v) potassium ioide-10% (m/v) ascorbic acid and filled to 10 mL with deionized water. Arsenic was determined by HG-AFS after 30 min. The concentration of As in water were expressed as µgAs/L or ppb. The Trace Elements in Water standard reference material (SRM 1643e, National Institute of Standards and Technology, Gaithersburg, MD, USA) containing 60.4 ± 0.7 µgAs/L was used for quality control. The concentrations of F^−^ were determinate by a potentiometric method using the ion selective electrode (Orion 9609) [23]. Briefly, calibration curves were prepared using standard solutions of NaF at final concentrations of 0.1 to 10 mgF^−^/L. An aliquot of water sample was diluted 1:1 with total ionic strength adjustment buffer in polyethylene tube. The electrode was immersed for 3 min and the potential (mV) of each sample was recorded. The mV readings were linear against the logarithm of F^−^ concentration mg/L (slope= −60, *r* = −0.998). The concentration of F^−^ in water was expressed as mgF^−^/L or ppm. The pH in water samples was determined by a potentiometric method.

### 2.6. Analysis of Arsenic in Urine

Urine aliquots were periodically shipped in dry ice by a 2-day mail service to the Nutrition Department at the University of North Carolina and analyzed in 1–2 weeks after delivery. The metabolites of iAs, including total iAs (iAsIII + iAsV), total methyl-As (MAs = MAsIII + MAsV) and total dimethyl-As (DMAs = DMAsIII + DMAsV) were measured by hydride generation (HG)-cryotrapping (CT)-atomic absorption spectrometry (AAS) as previously described [24]. This method cannot detect the non-toxic organic forms of As such as those found in seafood (e.g., arsenocholine or arsenobetaine) [25]. Thus, only As species associated with iAs exposure and metabolism were quantified. The NIST standard reference material SRM 2669 level II (Arsenic Species in Frozen Human Urine) was used for quality control during the analyses of urines from each of the shipments. The amounts of As species determined in the reference urine by HG-CT-AAS ranged from 91% to 106% (total iAs), 87% to 97% (MAs) and 89% to 99% (DMAs) of the corresponding certified values, and had a coefficient of variation <9%. The urinary concentrations of As species and the total speciated As (U-tAs = iAs + MAs + DMAs) were expressed in ng As/mL. To compensate for variations in dilution of urine (hydration status), the concentrations of As species and U-tAs were adjusted for specific gravity (SG), which was determined in freshly collected urine by pycnometer.

### 2.7. Analysis of Fluoride-in Urine

Additional urine aliquots were analyzed for F^−^ levels in Chihuahua University applying a potentiometric method using the ion selective electrode (Orion 9609) [26]. Analysis of certified urine fluoride reference materials F1110 and F1216 (Quebec Centre for Toxicology) containing 24.9 and 118 µmol F^−^/L (1.31 and 6.21 mg/L, respectively) was used for accuracy and precision control. The method accuracy ranged from 98% to 105% with the coefficients of variation of 2.1 to 5.2% for 6 samples. 

### 2.8. Statistical Analysis

Standard descriptive analyses were carried out using means and standard errors for continuous variables and frequencies for categorical variables. The distributions of continuous variables were also examined graphically using histograms and normal probability plots. Measurements of U-tAs and U-F^−^ were approximately log-normally distributed, and for these we report the geometric mean and standard deviation. All statistical analyses were performed using procedures available in Prism version 4.0. Associations of U-tAs and U-F^−^ with measures of As or F^−^ in water were analyzed by Spearman’s correlation coefficient (r_s_). 

## 3. Results and Discussion

### 3.1. Characteristics of the Study Population 

The basic characteristics of the study population, including gender, age, and indicators of iAs and F^−^ exposure (concentrations of As and F^−^ in water and urine) for male and female subjects are provided in Table 1. 

**Table 1:** Basic characteristics of the Chihuahua study population.

Characteristics	Alls N (%)	Males N (%)	Females N (%)
**Age (years)**			
18–90	1119 (100%)	370 (33.1%)	749 (66.9%)
18–40	457 (40.8%)	118 (10.5%)	339 (30.3%)
>40–50	235 (21.0%)	64 (5.7%)	171 (15.3%)
>50–65	281 (25.1%)	103 (9.2%)	178 (15.9%)
>65	146 (13.1%)	82 (7.3%)	64 (5.7%)
**Arsenic in water (µg/L)**			
<10	187 (16.7%)	54 (4.8%)	133 (11.9%)
>10–50	401 (35.8%)	137 (12.2%)	264 (23.6%)
>50–100	321 (28.7%)	106 (9.5%)	215 (19.2%)
>100	210 (18.8%)	73 (6.5%)	137 (12.2%)
**Fluoride in water (mg/L)**			
<0.5	184 (16.4%)	52 (4.6%)	132 (11.8%)
>0.5–1.5	471 (42.1%)	146 (13.1%)	325 (29.0%)
>1.5–3.0	222 (19.8%)	86 (7.7%)	136 (12.1%)
>3.0	242 (21.6%)	86 (7.7%)	156 (13.9%)
**Urinary Arsenic (ng/mL ^a^)**			
<35	365 (32.6%)	92 (8.2%)	273 (24.4%)
35–70	281 (25.1%)	102 (9.1%)	179 (16.0%
>70–140	314 (28.0%)	117 (10.4%)	197 (17.6%)
>140	159 (14.2%)	59 (5.3%)	100 (8.9%)
**Urinary Fluoride (µg/mL ^a^)**			
<1	243 (21.7%)	42 (3.7%)	201 (18.0%)
1–2	367 (32.8%)	100 (8.9%)	267 (23.9%)
>2–4	335 (29.9%)	142 (12.7%)	193 (17.2%
>4	174 (15.5%)	86 (7.7%)	88 (7.8%)

^a^ Urine adjusted for specific gravity.

### 3.2. Co-occurrence of Arsenic and Fluoride in the groundwater

**Figure 1:**
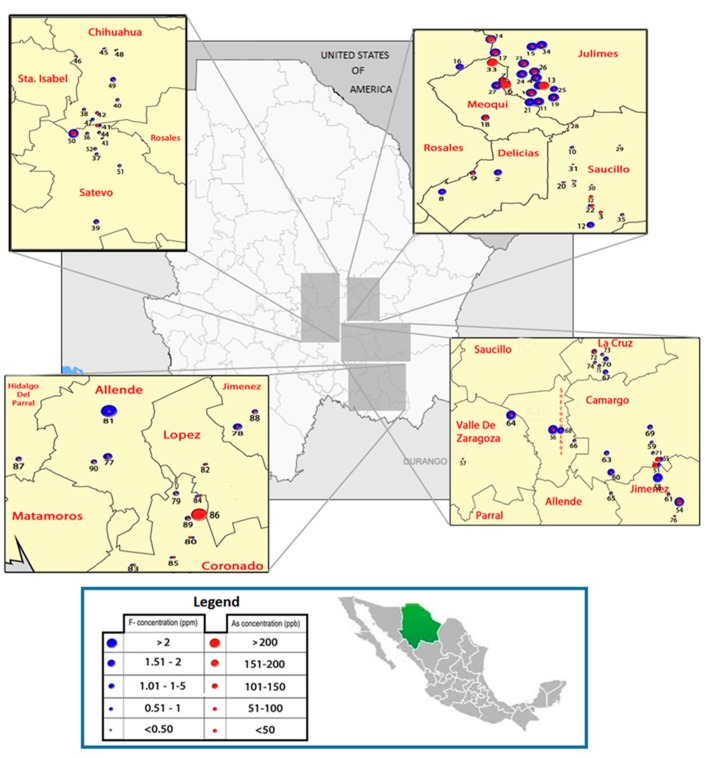
Distribution of arsenic and fluoride concentration in drinking water sources in the study area in Chihuahua, Mexico.

**Figure 2:**
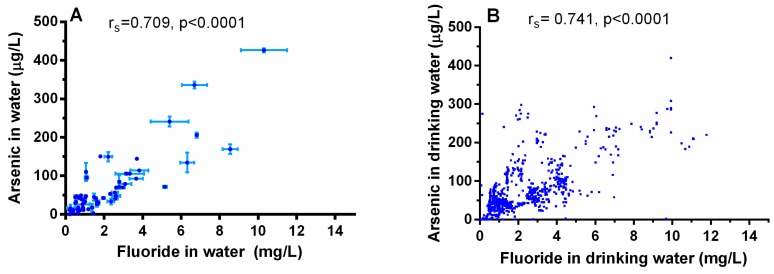
Correlation between arsenic and fluoride levels in drinking water: (**A**) Spearman correlation for water samples obtained from tube wells (3–6 samples per location, bars mean the minimum and maximum concentration of each location, *n* = 94 locations); and (**B**) from participant’ homes (1 sample per participant, *n* = 1119).

Most of the samples of water collected in this study (97%) contained detectable amounts of both As and F^−^ (Figure 1). The concentrations of As and F^−^ in tube water ranged from 0.1 to 419.8 µg/L and 0.05–11.8 mg/L, respectively. Well water from 77 of the 94 Chihuahua localities included in this study (*i.e.*, 81.9%) contained As at levels higher than the MCL value of 10 µg/L; for 33% As levels exceeded 50 µg/L. The concentrations of F^−^ were higher than WHO MCL of 1.5 mg/L in 37.2% locations; F^−^ levels in 30.9% of water samples were between 0.5 to 1.0 mgF^−^/L (the range recommended for maximum protection from dental caries) and 13.8% of samples contained F^−^ at concentration lower that the recommended levels. A positive, statistically significant correlation was found between the concentrations of As and F^−^ in tube-well water samples (r_S_ = 0.709, *p* < 0.0001, Figure 2A). A similarly strong correlation was between As and F^−^ levels in drinking water samples collected in subjects’ homes (r_S_ = 0.741, *p* < 0.0001, Figure 2B). The pH of the water samples ranged from 6.38 to 8.51, with a mean ± SD value of 7.75 ± 0.35, 96.1% of samples were higher than pH 7. Spearman tests showed a positive statistically significant correlation between water iAs and water F^−^ concentrations with pH value (r_s_ = 0.795 and 0.824, respectively).

Because the concurrent exposure to As and F^−^ in drinking water is a relatively common phenomenon, the co-occurrence of these two contaminants and potential adverse effects of the co-exposure are of great importance for human risk assessment [2,27,28]. In the present study, we found a co-occurrence of As and F^−^ in drinking water supplies in the southwest part of Chihuahua Mexico. Geological faults present in the Chihuahua are believed to be the conduit for the iAs and F^−^ found in the local groundwater supplies [28]. These parallel faults stretch in the northwest-southeast direction for up to 200 km and cover most of the study area. The presence of these contaminants in the groundwater of Chihuahua Mexico has also been attributed to geogenic sources [29,30]. Specifically the release of iAs from the volcanic glass present in rhyolite and tuffs deposits in and surrounding alluvian aquifer system, and the presence of shales that outcrop in the sediments of this area; fluorapatite is also present in the rhyolites and the volcanic rocks [29].

Some studies have also attributed high F^−^ levels in the As-contaminated groundwaters under reducing environments, such as an acidic pH, to the weathering of F^−^bearing minerals such as fluorite and/or fluorapatite [31]. However, groundwater from Chihuahua region is predominantly oxidizing with neutral to a slightly alkaline pH. Our data on pH and other parameters in 35 groundwater samples from the study are, including electrical conductivity and redox potential, also indicate the oxidizing conditions [30]. Because of the increasing use of groundwater in the semiarid area of the study, the dissolved iAs and F^−^ under reducing conditions could move upward to shallow groundwater where iAs and F^−^ remain in solution in oxidized conditions [30,32]. Consistent with previous reports [28,29,30], we found wide ranges of iAs and F^−^ concentrations in the tube well water samples collected in several semirural localities in this area (0.1 to 419.8 µgAs/L and 0.01–11.8 mg F^−^/L). The strong positive correlations between As and F^−^ levels in the well water (r_s_ = 0.709; *p* < 0.0001) and the household water samples (r_s_ = 0.741; *p* < 0.0001) suggests that a similar association must exist between the geological sources of both minerals in the study area, or that both come from the same source.

### 3.3. Co-Occurrence of Arsenic and Fluoride in the Urine

**Figure 3:**
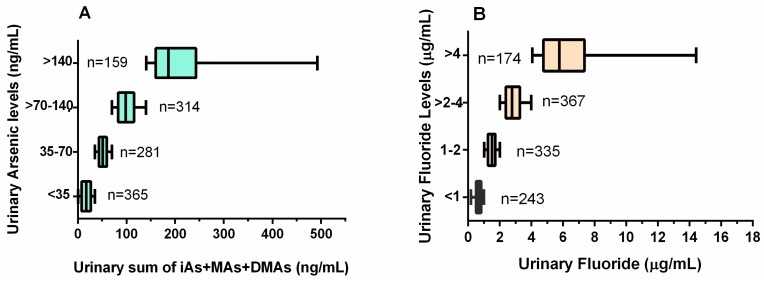
The distribution of total speciated arsenic (**A**) and fluoride levels (**B**) in the urine samples—stratified by 1×, 2× and 4× Biological Exposure Index value. Median values (vertical lines) and the interquartile range (boxes) are shown. The whiskers represent minimum and maximum values.

**Figure 4:**
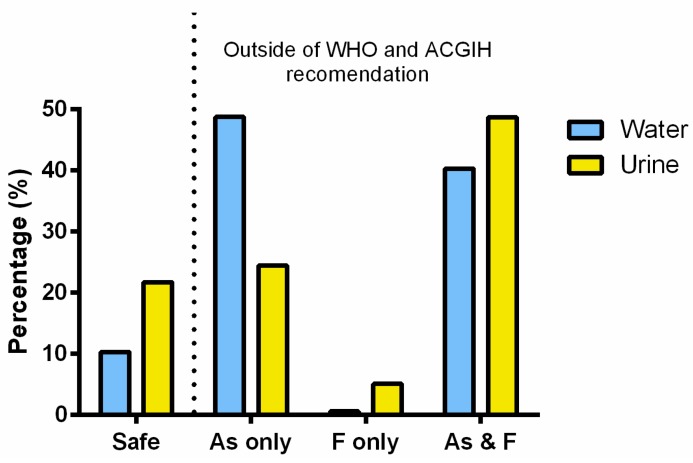
The percentage of water samples with arsenic and/or fluoride levels below or above the WHO maximum contaminant level of 10 µgAs/L and 1.5 mgF^−^/L, respectively. The percentage of urine samples with the total speciated arsenic and/or fluoride levels below or above the Biological Exposure Index value of 35 ng/mL and 2 µg/mL, respectively, established by American Conference of Governmental Industrial Hygienist (ACGIH).

Both As and F^−^ were detected in the urine samples collected from the study participants. The concentrations of U-tAs ranged from 0.5 to 467.9 ng As/mL (Figure 3A). The concentrations of U-F^−^ ranged from 0.1 to 14.4 µgF^−^/mL (Figure 3B). We stratified the levels of U-tAs from participants into four groups based on the Biological Exposure Index (BEI) of 35 ng/mL, which is used in the practice of industrial hygiene as guidelines for occupational exposure to iAs [33], and represents conditions under which the worker may be repeatedly exposed without adverse health effects. The distribution of U-tAs and U-F^−^ was evaluated using four concentration ranges: <35, 35–70, >70–140, >140 ng/mL defined by 1×, 2× and 4× BEI value. Notably, 67% of samples contained U-tAs at the concentrations higher than the BEI value (Figure 3A). Similarly, U-F^−^ concentrations were stratified in four concentration ranges (<1, 1–2, >2–4, >4 µg/mL) based on the BEI of 2 µg/mL [34]. Almost 49% of urine samples contained F^−^ at levels higher than the BEI value (Figure 3B). Only, 21.7% of urine samples were below BEI values for both U-tAs and U-F^−^; 24.7% were above the BEI only for U-tAs; 5.1% above the BEI only for U-F^−^, and 48.7 % exceeded the BEI values for both U-tAs and U-F^−^ (Figure 4).

**Figure 5:**
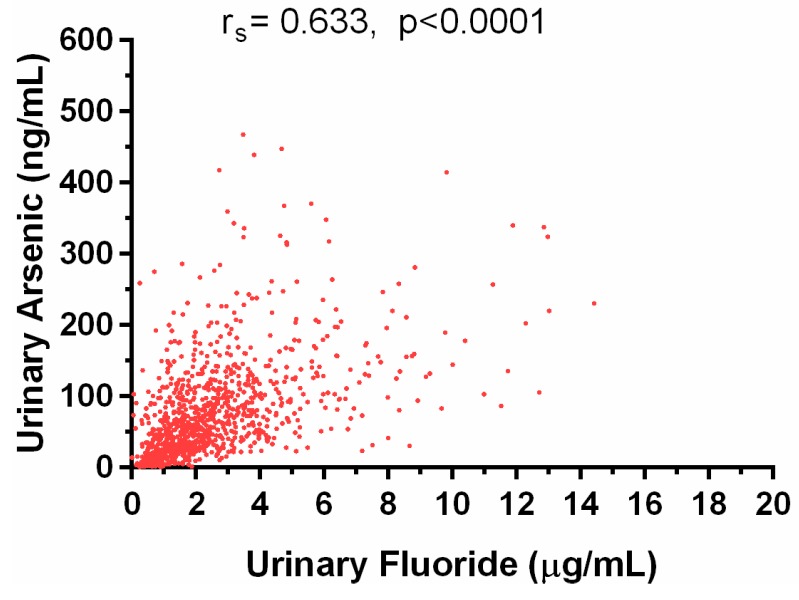
The association between urinary arsenic and fluoride concentrations adjusted for specific gravity. Spearman correlation coefficient and p value are shown for 1119 samples.

Importantly, we found that a high proportion of our study participants (>48%) had excessive (>BEI) levels of both U-tAs and U-F^−^. We found a positive statistically significant correlation between the levels of U-tAs and U-F^−^ (r_s_ = 0.633; *p* < 0.0001; Figure 5), suggesting that the Chihuahua residents who are exposed to high iAs levels are also likely to be exposed to potentially hazardous levels of F^−^. In addition, significant differences were found in U-tAs and U-F^−^ levels between males and females. Specifically, urine from women contained on average less tAs (41.5 *vs* 59.4 ng/mL) and F^−^ (1.9 *vs* 2.4 µg/mL) than urine of men. 

### 3.4. Relationship between Arsenic and Fluoride Levels in the Water and the Urine

**Figure 6:**
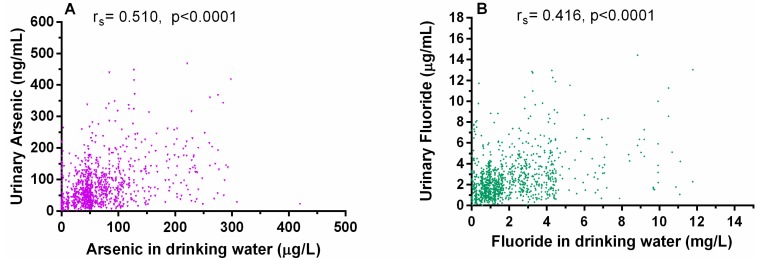
Correlations between arsenic and fluoride concentrations in water and urine: (**A**) Positive correlation between urinary arsenic and drinking water arsenic; (**B**) Positive correlation between urinary fluoride and drinking water fluoride. Urinary arsenic and fluoride concentrations were adjusted for specific gravity. Spearman correlation coefficient and p value are shown for 1119 samples.

Positive statistically significant correlations were found between U-tAs and water subjects’ homes iAs concentrations (r_s_ = 0.510; *p* < 0.0001; Figure 6A) and between urinary and water F^−^ concentrations (r_s_ = 0.416; *p* < 0.0001; Figure 6B).

The analyses of urines collected in the Chihuahua study area provide evidence that drinking water is a major source of human exposure to both iAs and F^−^. The ranges of U-tAs (0.5 to 467.9 ngAs/mL) and U-F^−^ concentrations (0.1 to 14.4 µg F^−^/mL) are consistent with wide ranges of the exposure. Moreover, the correlation between U-tAs and U-F- is strong (r_s_ = 0.633, *p* < 0.001). Another common source of iAs and F^−^ exposure is diet, including foods rich in iAs (e.g., rice, fruits, fruit juices or food cooked iAs contaminated water) [35,36] and tea, maize, cereals, juices and sodas prepared with high F^−^ water, which are known to contain substantial amounts of F^−^ [37,38]. Dental care products (e.g., toothpastes or mouth washes) are also a likely source of F^−^ for Chihuahua population. The Food and Agriculture Organization of the United Nations (FAO)/WHO established the provisional tolerable weekly intake (PTWI) value based on the provisional tolerable daily intake (PTDI) for iAs of 2 µgAs/Kg body-weight (bw) per day [39]. The minimal risk level for daily oral F^−^ intake, 0.05 mg/Kg per day [4], has been based on the non-observable adverse effect level (NOAEL) of 0.15 mg F^−^/Kg/day for an increased bone fracture rate. Given the water concentrations of iAs and F^−^ determined in this study and assuming the total daily water intake of 0.5 to 3 L, it is obvious that iAs and F^−^ intakes by most of the study participants exceeds the established safe levels.

The chronic exposure to high levels of iAs and F^−^ in drinking water represents a significant health risk for residents in the southwest part of Chihuahua. Results of the present study show that 81.9% of subjects drink water with As concentration above the MCL value of 10 µg/L; U-tAs concentration in 67% of samples exceeded the BEI value of 35 ngAs/mL. Similarly 37.2% of water samples contained F^−^ at concentrations higher that the WHO MCL of 1.5 mg/L and F^−^ concentration in 49% of urine samples was higher than the BEI value of 2 µgF^−^/mL. We used as reference in urine, the BEI value because even it is used in the practice of industrial hygiene as guidelines for occupational exposure, it is the only newly updated reference value for regulation of iAs and its methylated metabolites in urine, and F^−^ level in urine.

### 3.5. Risk Associated with the Combined Exposure to Inorganic Arsenic and Fluoride

The focus of the present study was to characterize exposures to iAs and F in the Chihuahua cohort. Our future studies will systematically evaluate potential adverse affects of the iAs/F^−^ co-exposure in this cohort. Notably, both signs of dental fluorosis and skin lesions typical of the chronic iAs exposure (keratosis and changes in pigmentation) were observed in several participants during the introductory medical exam (unpublished data). Previously, dental fluorosis was reported for 80% of the population in this area as a consequence of the high F^−^ levels in drinking water (0.7 to 8.6 mg/L) [40]. Although the adverse effects of the isolated exposures to iAs and F^−^ have been widely studied and are relatively characterized, the potential effects associated with the simultaneous exposure have not been systematically examined. Thus, more studies are needed to better characterize the health risks associated with the combined exposures to iAs and F^−^ in the geographical regions where both iAs and F^−^ are present in drinking water supplies. Our results show that Chihuahua, where the majority of ~3.5 million residents rely on groundwater as the main drinking water supply and where alternative sources of clean water are scares, is one of these regions. 

## 4. Conclusions

Results of this study suggest that a significant number of residents in the southwest part of Chihuahua are chronically exposed to high levels of both iAs and F^−^ in drinking water. This co-ocurrence should be taken in account by local authorities when choosing and installing the water purification systems. The U-tAs and U-F^−^ at levels exceeding the ACGIH established values were found in 48.7% of the subjects participating in this study. Thus, a systematic testing of drinking water supplies, including individual private wells, for As and F^−^ should be a top priority for the local government and public health authorities. Antagonistic and synergistic effects, or a lack of any interaction have been reported in the co-ocurrance of iAs and F^−^ [16]. For example, it has been suggested that there are no interactions between the absorption and excretion of As and F^−^ in humans after ingestion of drinking water with naturally high concentrations of both iAs and F^−^ [41]. But, both synergistic and antagonistic interactions between iAs and F^−^ appear to be possible, depending on the exposure level and specific physiological/metabolic processes or endpoints. Given the potential of adverse health effects, immediate measures should be taken to reduce the exposure, particularly for vulnerable population, and specifically for pregnant women and children. The role of F^−^ exposure in the health risks previously attributed to iAs exposure alone [19,20] should be systematically studied. 
